# Green Manure Species for Phytoremediation of Soil With Tebuthiuron and Vinasse

**DOI:** 10.3389/fbioe.2020.613642

**Published:** 2021-01-05

**Authors:** Luziane Cristina Ferreira, Bruno Rafael de Almeida Moreira, Renato Nallin Montagnolli, Evandro Pereira Prado, Ronaldo da Silva Viana, Rafael Simões Tomaz, Jaqueline Matos Cruz, Ederio Dino Bidoia, Yanca Araujo Frias, Paulo Renato Matos Lopes

**Affiliations:** ^1^Department of Plant Production, College of Technology and Agricultural Sciences, São Paulo State University (UNESP), Dracena, Brazil; ^2^Department of Natural Sciences, Mathematics and Education, Agricultural Sciences Center, Federal University of São Carlos (UFSCar), Araras, Brazil; ^3^Department of Biochemistry and Microbiology, Biosciences Institute, São Paulo State University (UNESP), Rio Claro, Brazil

**Keywords:** bioremediation, ecotoxicity, fertigation, herbicide, sugarcane

## Abstract

Tebuthiuron is often used to control weed growth in sugarcane cultures. This herbicide is highly toxic and can persist in soil for up to 2 years according to its degradation half-life. Hence, its residual effect is highly hazardous for the environment and local habitants via leaching, surface runoff. Screening out of species of green manure as potential phytoremediators for tebuthiuron in soil, with and with no vinasse, accordingly is the scientific point of this study. Green manure species selected for the trial in greenhouse were jack bean [*Canavalia ensiformis* (L.) DC.], pigeon pea [*Cajanus cajan* (L. Millsp.)], velvet bean [*Mucuna pruriens* (L.) DC.)], and millet [*Pennisetum glaucum* (L.) R.Br.], and *Crotalaria juncea* L. as bioindicator of this herbicide. The determination/quantification of height, stem diameter, and number of leaves in all plants were monitored, as well as other morphological traits for drafting any inference on biomass production. Moreover, ecotoxicity bioassays were performed from soil samples at the beginning and at the end of the experiment. Results showed preliminary evidence of effective phytoremediation capacity by *M. pruriens* and *P. glaucum* in soils with tebuthiuron, as the growth of *C. juncea* was sustained. Both Gompertz approach and principal component analysis predicted that these green manure species could grow healthier and for longer periods in soils containing tebuthiuron and vinasse and, thus, reduce physiological anomalies due to ecotoxicity. The implications of this study may aid in the implementation of cost-effective strategies targeting decontamination of tebuthiuron in sugarcane crops with vinasse application in fertigation.

## Introduction

Sugarcane is affected by weeds, despite its highly efficient photosynthetic pathway (C_4_) that promotes adequate development, especially in its early stages. Weeds compete for available soil resources and therefore undermine agricultural yields (Victoria Filho and Christoffoleti, 2004; Sandaniel et al., 2008).

The planting of sugarcane takes place in wide open areas so that high productivity is achieved, aided by technological tools for the proper weed management as herbicides (Kuva et al., 2008; Oliveira and Brighenti, 2011). Such chemical method is the first choice of agricultural producers due to its ease of access, availability, and low operational costs, compared to other control techniques (Kuva et al., 2008).

Among the herbicides commercialized for sugarcane, tebuthiuron is the most used, whose selective pre-emergent action controls main weeds in the crop (Moraes et al., 2016). This molecule [1-(5-tert-butyl-1,3,4-thiadiazol-2-yl)-1,3-dimethylurea] has a systemic action and acts in the inhibition of photosystem II (Breitenbach et al., 2001). However, tebuthiuron can cause environmental damage since it is considered dangerous to the environment due to its high persistence and long half-life in the environment, moderate to extreme toxicity (Rodrigues and Almeida, 2011), low sorting capacity in soil (Koskinen et al., 1996), and high solubility in water (Franco-Bernardes et al., 2014). Hence, residual concentration is an extremely important factor, as it results in a greater potential for contamination. Therefore, successive applications without proper management can make its potential for impact even greater on soil and groundwater (Christofoletti et al., 2017).

Vinasse can also be applied to farmlands as growth-inducing agents, as opposed to herbicides. Vinasse is one of the many by-products of sugar production also found in alcohol distilleries in enormous quantities. It is highly applicable as fertilizer during crop production (Andrade, 2007). It is considered a residue from the alcohol production, generated at a 10–14:1 ratio (Assad, 2017). The outstandingly large amount of vinasse generated everyday highlights its expressive polluting potential and, therefore, demands the development of proper disposal protocols. However, vinasse can also be used to enrich soils due to its nutritional value. Still, its environmental effects combined with herbicides are yet to be determined.

The cleaning up process of areas with previous pesticides release is not simple, but fortunately, many solutions have been improved in the past decades. Feasible solutions should follow four requirements, as proposed by Ferro et al. (1994): (i) high decontamination efficiency, (ii) straightforward execution, (iii) fast and reliable protocols, and (iv) cost effectiveness. Bioremediation is as an ecologically viable strategy that meets such requirements during the treatment of impacted areas by organic pollutants. The acceleration of natural biological processes that reduce the concentration and toxic effects of polluting agents is the core of all bioremediation strategies (Fasanella and Cardoso, 2016).

Phytoremediation further expands this definition by using plants to reduce the toxicity of contaminants in the environment (Ali et al., 2013). Research related to this technique seeks to understand the plant–contaminant interactions that may lead to full pollutant removal (Vasconcellos et al., 2012). Therefore, the plants must be capable of absorbing toxic elements in the soil to promote decontamination (Souza et al., 2011).

Pires et al. (2008) reported that millet (*Pennisetum typhoides*), velvet bean (*Stizolobium aterrimum*), Jack bean (*Canavalia ensiformis*), and pigeon pea (*Cajanus cajan*) were highly effective toward tebuthiuron phytoremediation. They used sunn hemp (*Crotalarea juncea*) as the bioindicator plant. Several studies reinforce this approach, as many authors have observed a decrease in pesticides concentration in soils by using phytoremediation (Pires et al., 2003, 2005, 2006; Pires et al., 2008; Madalão et al., 2013; Melo et al., 2017).

However, there is a major drawback in all those studies: the toxicity of these samples has not been quantified before and after the treatments. The degradation of organic compounds could potentially generate intermediate compounds that are often more toxic than the original formulation (Rocha et al., 2018). We argue that it is imperative to evaluate the ecotoxicological potential in a broader time-dependent approach to demonstrate the success of bioremediation strategies (Banks and Schultz, 2005).

In this context, we evaluated the potential of four plant species to remediate soil samples contaminated with tebuthiuron and the effects of vinasse in the process.

## Materials and Methods

Experiments were set up in a greenhouse located at the College of Agricultural and Technological Sciences, Sao Paulo State University (Unesp), Dracena, São Paulo, Brazil, with geographical coordinates of 21°28′57″ S 51°31′58″ W and 400 m elevation.

According to the Köppen (1948) classification, the regional climate type is Aw (tropical humid). The average local temperature and precipitation are 22.1°C and 1,200 mm, respectively. The meteorological data were provided by our own station (Dracena EMA/FCAT).

The observations occurred between May and July 2019. The average temperature and relative humidity were 22.6°C and 62.9%, respectively, also obtained from the Dracena EMA/FCAT weather station.

### Soil, Vinasse, and Tebuthiuron Sampling

The regional soil is a dystrophic red-yellow argisol type according to the classification proposed by Santos et al. (2018). The physical analysis revealed that it is composed of 89.9% sand, 7.1% clay, and 3.0% silt.

The soil has the following chemical characteristics: phosphorus, 5 mg/dm^3^; organic matter, 3 mg/dm^3^; pH 5.2; potassium, 1.7 mmolc/dm^3^; calcium, 15 mmolc/dm^3^; magnesium, 4 mmolc/dm^3^; H + Al, 13 mmolc/dm^3^; CTC, 34 mmolc/dm^3^; sum of bases, 21 mmolc/dm^3^; and base CTC saturation (V%), 61%. This characterization served as a basis to the optimal fertilizing conditions in our pots for all the proposed species.

Fertilizer dosages per pot were set individually to meet each species needs. We applied 80 g of urea diluted in 1.5 L of water, divided into three applications, in *Pennisetum glaucum* (L.) R.Br. We applied 8 g of urea diluted in 4.5 L of water, applied only once at sowing, in legumes [*C. ensiformis* (L.) DC., *C. cajan* (L. Millsp.), *Mucuna pruriens* (L.) DC.]. For all other pots, we added 125 g of KCl diluted in 6 L of water, divided into three applications, and 445 g of simple super phosphate to 320 L of soil, necessary for filling the pot total volume. The volume of each vessel was 4 dm^3^.

The vinasse was collected from a sugar-energy plant in the Dracena-SP region using sterile glass bottles. The vinasse was subsequently stored in a refrigerator at 4°C until its use in the experimental units preparation (stored for 3 days).

The herbicide tebuthiuron was provided by Combine® 500SC—Dow AgroSciences Industrial Ltd.

### Plant Species

The plant species were chosen according to their capability to remediate pesticide-contaminated soils. Their agricultural potential to improve overall soil quality was another criterion. We narrowed our plants selection to species that are often found as green manure and/or forage in the rotation of the sugarcane cultures. Thus, we used the following species: pigeon pea (*C. cajan*), jack bean (*C. ensiformis*) (Madalão et al., 2013, 2016), velvet bean (*M. pruriens*) (Pires et al., 2005, 2008), and millet (*P. glaucum*) (Pires et al., 2008).

### Experimental Setup

We designed the experiments according to a randomized blocks approach at a 2 × 2 × 4 factorial scheme with five repetitions. The parameters were tebuthiuron concentration, vinasse volume, and the four plant species.

The treatments are further referred to in this paper as indicated in the brackets: [T–], absence of tebuthiuron; [T+], presence of tebuthiuron; [V–], absence of vinasse; and [V+] presence of vinasse.

### Preparation of Experimental Units

Experimental units (pots 4 dm3 and their contents) were filled with soil and received the four potentially phytoremediating species. Treatments containing vinasse had to undergo manual compound application to endure homogeneity. The vinasse was added first, at 150 m^3^ ha^−1^ (150 ml dm^−3^), following the CETESB Technical Standard P4.231/2005 (2005) on the procedures to apply vinasse to agricultural soils.

The Combine® 500 SC was sprayed at the following day, at 2 L ha^−1^ as the recommended rate of this herbicide in sugarcane crops, using a CO_2_ pressurized sprayer (Herbicat®) equipped with six XR 8002 flat jet nozzles at a pressure of 2 bar (0.65 L min^−1^ flowrate) from a minimum distance of 0.5 m. The application was carried out 0.75 m above the pots at a constant speed (5 km h^−1^) until 250 L ha^−1^ had been applied. Environmental conditions such as temperature and relative humidity were monitored at the time of spraying using a portable digital thermo-hygro-anemometer-luximeter (Instrutherm® model THAL-300). The spraying occurred inside our greenhouse to avoid wind interference during the application. An equivalent volume of deionized water was added to treatments without vinasse and/or tebuthiuron.

Finally, 10 seeds per pot were sown the day after the tebuthiuron application. Thinning was performed on the eight DAS to keep only one plant per pot. These were irrigated daily by microsprinklers for 60 min (30 min at 6:00 a.m. and 30 min at 6:00 p.m.) to ensure adequate conditions for plant growth.

The cultivation of species with phytoremediation potential was performed for 50 days. Therefore, the final phytoremediation evaluation time for these plants is t_50_.

Ten days after harvesting the plants of these species, we sowed 10 seeds of the bioindicator species sunn hemp (*C. juncea* L). Thinning was performed on the 10th DAS. These were also irrigated daily by microsprinkling at 6 mm/h for 60 min (30 min at 6:00 a.m. and 30 min at 6:00 p.m.) to sustain proper plant development conditions.

### Evaluation of Plant Growth

Plant growth was quantified weekly. The monitored parameters were (i) stem diameter in millimeter, (ii) height of shoot in centimeter, and (iii) number of leaves.

For *C. cajan, C. ensiformis, M. pruriens*, and *P. glaucum*, the periodic monitoring running from the 15th to the 50th DAS yielded six time-point datasets until the end (t_50_) of cultivation. *C. juncea* was planted in all experimental units, and its morphological parameters were monitored from 17th to the 45th DAS, thus yielding five time-point datasets until the end of experiment (t_95_).

After cultivation, plants were separated for quantification of biomass: the fresh and dry matter of shoots and roots. The separation of the shoots and roots occurred by cutting the stalks close to the soil between the stem and the root. The roots were thoroughly washed so that all the soil was removed. After separation, each fraction was weighed separately and then packed in a paper bag to dryness in oven at 65°C, over 72 h. The resulting samples were weighted again to obtain the dry mass.

### Ecotoxicity Bioassays

Bioassays monitored the ecotoxicological potential of each treatment over the proposed time frame. The phytotoxicity of the soil samples was determined at the initial (t_0_) and final (t_50_) times when it was cultivated the phytoremediation species (*C. cajan, C. ensiformis, M. pruriens*, and *P. glaucum*), just before the *C. juncea* introduction.

Lettuce seeds (*Lactuca sativa*) were the test organism, according to Sobrero and Ronco (2004). Phytotoxic effect determination of each treatment was performed in six replicates from the solubilized soil extract, according to the NBR 10.006 (ABNT, 2004).

Ecotoxicity tests were prepared in Petri dishes with filter paper supported with 2.0 ml of the solubilized extract and 10 lettuce seeds. Petri dishes were then wrapped with polyvinyl chloride (PVC) film and incubated at 20 ± 2°C for 120 h in the dark.

Positive control was prepared using 0.05 M zinc sulfate to inhibit seed germination and negative control using deionized water to test the base germination and growth values of the seeds (Sobrero and Ronco, 2004).

The following parameters were determined: seed germination, root and hypocotyl elongation (≥0.1 mm), and the Germination Index (GI) that combines seed germination (% G) and root elongation (% R) at the CN. The GI was used to assess the toxicity of soil samples in the test organism, according to Equation 1 (Labouriau and Agudo, 1987):

(1) $$\begin{array}{r} G_{I}=\;\frac{G\times R}{100}. \end{array}$$

### Data Analysis

Procedures of Shapiro–Wilk and Bartlett checked the normalcy and homoscedasticity of the dataset, respectively, and the analysis of variance tested the significance of effect of factors on the soil phytoremediation. The tests were separated by *post hoc* Tukey's honest significant difference (HSD) test. The data of temporal variability were fitted for Gompertz (Equation 2). This stochastic model has probability density function enough to predict how long would it take the green manure in soil with tebuthiuron and stillage to reach the maximum of growth and develop and stop. The kinetic parameters, α, β, and κ, will assist in drafting inferences about the potential phytoremediators and figuring out the best chance to deal with how to solve the side effects of these contaminants as much suitably as possible.

(2) $$\begin{array}{r} f=\;\alpha e^{-\beta e^{-kx}} \end{array}$$

where:

f(*x*): height or stem diameter;

*x*: time of sampling;

α: upper asymptote or the maximum of height or stem diameter;

β: inflection point;

κ: specific-growth rate;

ακ*e*^−1^: absolute-growth rate;

*e*: Euler number.

An unbiased soft computing technique of contour plotting was performed to chart the spatial production of phytomass by the models for green manure. To an optimization of visualization of non-Boolean patterns in chromatic wireframe by contour plotting approach, fuzzy logic to turn any ambiguity off from the data was implemented. Another method of applying non-traditional mathematics to establish an eventual effect of green manure on the decontamination of the soil included principal component analysis (PCA). The Kaiser–Meyer–Olkin test was applied to determine how many components should be necessary to reduce the high-dimensionality data, while preserving as much attributable variability as possible into orthogonal subsets without collinearities. The software was R-project. This multiparadigm programming open-coding language provides a user-friendly environment for statistical computing and graphs.

## Results and Discussion

### Performance of Species of Green Manure

#### Morphological Traits

The effects of green manure species and the microenvironment toward the phytoremediation potential was determined. Phytoremediators response occurred regardless of their morphological traits. The variations found in each assay allowed us to determine their sources (Table 1).

**Table 1:** Morphological aspects of green manure species in soil with tebuthiuron and vinasse.

**Species**	**Test**			
	**T-V-**	**T-V+**	**T+V-**	**T+V+**
	**Height, cm**			
*C. cajan*	64.40 Aa	49.20 Bb	ND^*^	ND^*^
*C. ensiformis*	75.00 Aa	64.80 Bb	ND^*^	ND^*^
*M. pruriens*	125.80 Aa	134.60 Aa	115.50 Aab	44.00 Bb
*P. glaucum*	71.00 Aa	53.60 Ba	60.75 Ba	56.65 Aa
	**Diameter, mm**			
*C. cajan*	5.40 Ba	4.69 Bb	ND^*^	ND^*^
*C. ensiformis*	5.65 Ba	5.60 Bb	ND^*^	ND^*^
*M. pruriens*	5.05 Ba	5.05 Ba	4.45 Bab	3.50 Bb
*P. glaucum*	52.70 Aa	42.30 Aa	35.90 Aa	23.15 Ab
	**Leaves**			
*C. cajan*	16.80 Aba	13.00 Bb	ND^*^	ND^*^
*C. ensiformis*	4.80 Ca	4.20 Cb	ND^*^	ND^*^
*M. pruriens*	14.40 Ba	11.00 Ba	11.50 Ba	2.00 Ab
*P. glaucum*	22.50 Aa	20.80 Aa	20.50 Aa	16.30 Ba

*Same letters, whether uppercase in the column and lowercase in the row, describe statistically similar means by post hoc Tukey's HSD test at p < 0.05;*.

* *As the plant dead, the trait became undetected (ND)*.

**Figure 1:**
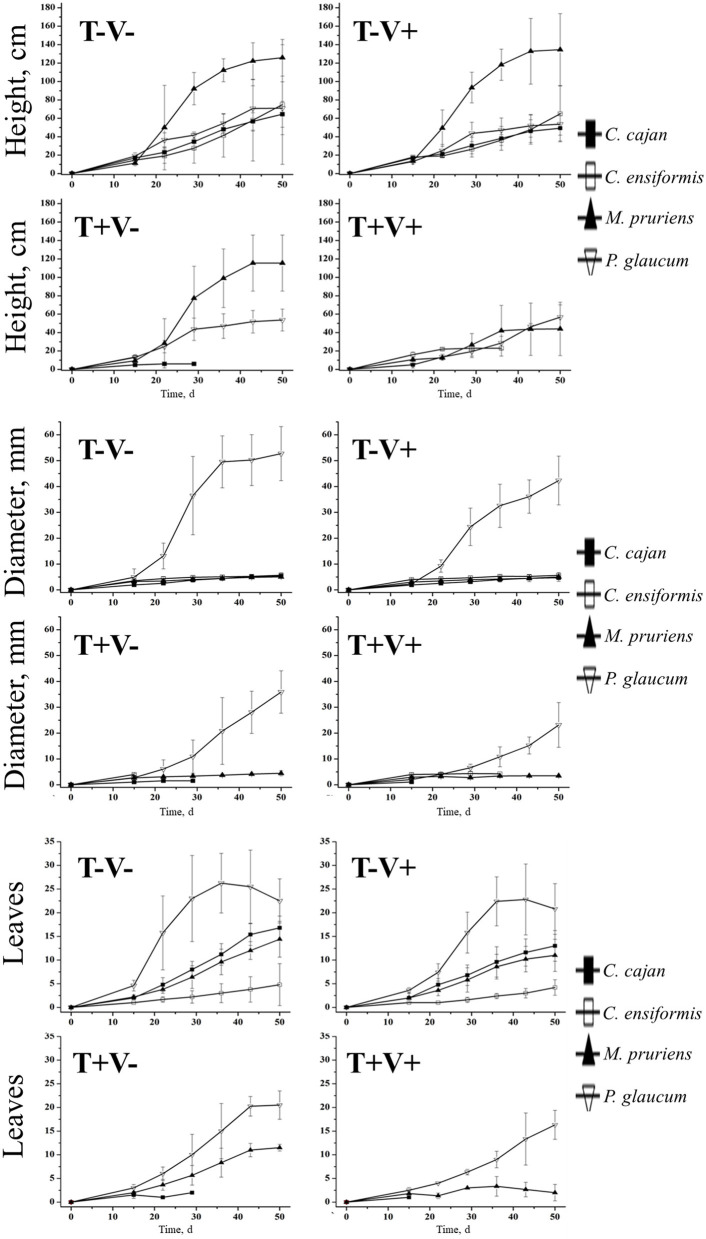
Production of biomass by green manure species in soil with tebuthiuron and vinasse.

Tebuthiuron and vinasse underwent reactions in the soil, thus collectively influenced the phytotoxicity of the microclimate. The herbicide alone was more toxic to *C. cajan* and *C. ensiforms*. In contrast, *M. pruriens* and *P. glaucum* resisted longer to chemically stressed microenvironment. Vinasse addition significantly reduced the toxicity. Hence, green manure species produced larger amounts of mass of roots and shoots in these soil samples (Figure 1).

Advantages of vinasse on the ecotoxicity were more prominent in *M. pruriens* and *P. glaucum*. These were the most effective strategies of manuring for phytoremediation potential. The primary assumption for vinasse attenuation by on phytotoxicity of soil may be its availability of soluble carbon. Thus, it is likely to power up the microbial metabolism and enhance the subsequent degradation of pesticides (Prata et al., 2000, 2001; Villaverde et al., 2008). Tebuthiuron is highly available and can move smoothly through the structure of agricultural soils with lows levels of organic matter and clay (Chang and Stritzke, 1977). These authors reported 40.00 and 1.00% herbicide adsorbed in particles at 4.8 and 0.30% organic matter, respectively. Bioavailability is one of the keys to an effective and consistent biodegradation. If the pollutant or contaminant is not available from the environment, (micro)organism cannot successfully perform longer. The nature and physicochemical properties of the pesticide (e.g., chemical stability, spatial structure, feedback effect, and intermediate metabolites) and its multiplicity of interactions with the rhizosphere are factors influencing greatly its bioavailability and, of course, kinetics of biodegradation.

#### Kinetics of Growth and Development

**Figure 2:**
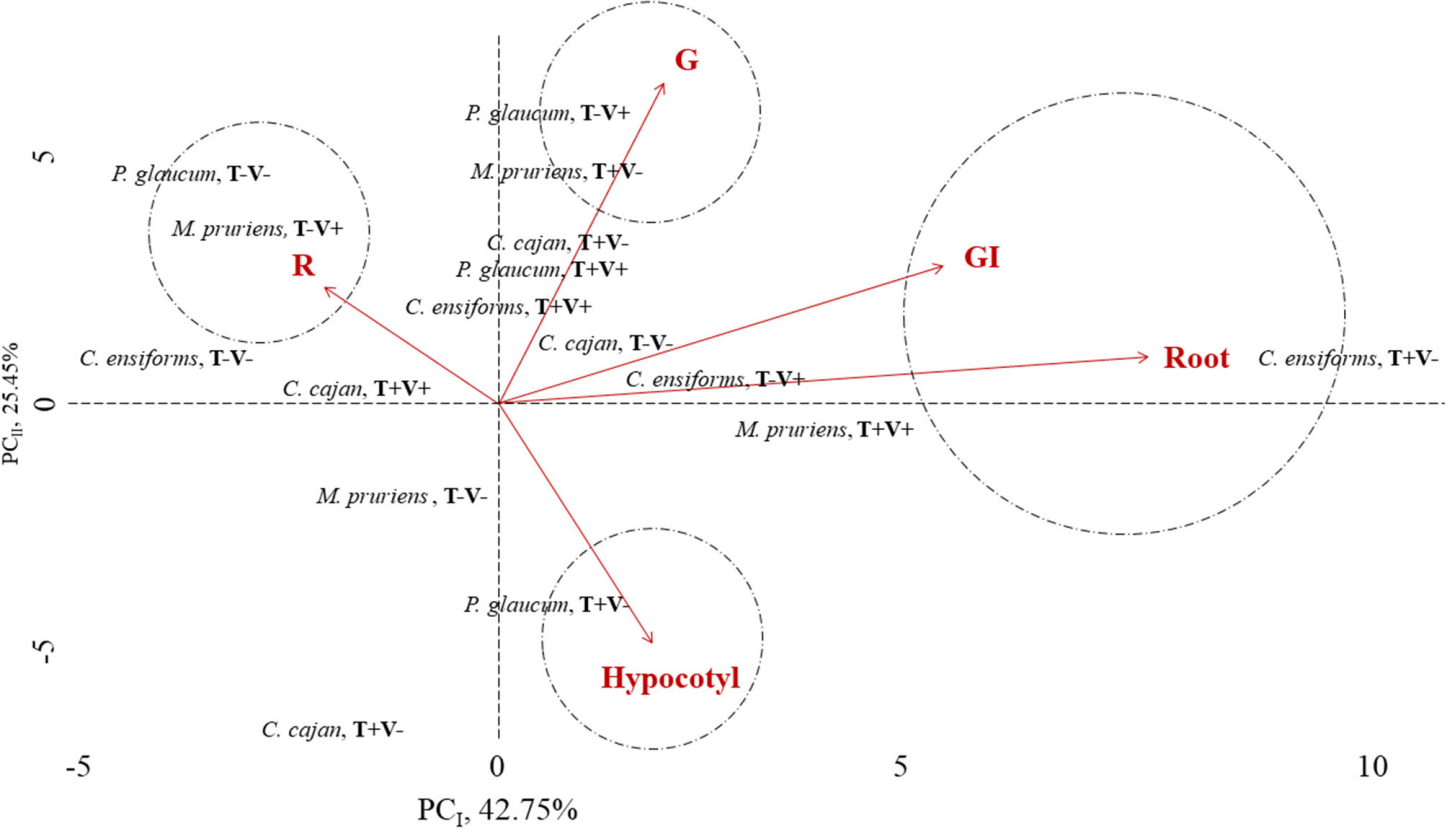
Kinetics of growth and development of green manure species in soil with tebuthiuron and vinasse.

The Gompertz approach predicted accurately how long would it take for the chemical contamination of soil by tebuthiuron to become limiting for the growth and development of green manure species (Figure 2). Estimates for the absolute primary growth rate for the *C. cajan* in soil with tebuthiuron and vinasse combined was the lowest (Table 2). As long as the target molecule is rather persistent than readily degradable, the more probable the strategy of manuring is to inefficiently decontaminate an area. The highest estimations for both the maximum of size and absolute growth rate for this potential phytoremediator in soil with no tebuthiuron supported the high phytotoxicity of this herbicide to the primary growth.

**Table 2:** Kinetic parameters for the growth and development of green manure species in soil with tebuthiuron and vinasse.

**Species**	**Test**	**Parameter**				**${\text{R}}_{\text{adj}}^{2}$**
		**α**	**β**	**κ**	***ακe^**−1**^***	
		**Height**				
*C. cajan*	T-V-	78.50	4.40	0.40	11.55	0.9895^**^
	T-V+	61.65	4.50	0.45	10.20	0.9585^*^
	T+V-	5.10	560.65	4.80	9.00	0.0350
	T+V+	5.05	1.05	1.45	2.70	0.9995^**^
*C. ensiformis*	T-V-	217.90	4.70	0.20	16.05	0.9895^**^
	T-V+	193.05	4.20	0.20	14.20	0.9710^*^
	T+V-	4.50	3.20e^−7^	−5.20	−8.60	0.2285
	T+V+	16.85	730.90	4.80	29.75	0.9995^**^
*M. pruriens*	T-V-	127.95	18.25	1.00	47.05	0.8755^*^
	T-V+	140.40	14.40	0.90	46.50	0.8495^*^
	T+V-	121.80	24.95	0.95	42.55	0.7815^*^
	T+V+	50.85	7.45	0.60	11.20	0.7405^*^
*P. glaucum*	T-V-	80.30	4.55	0.50	14.75	0.8190^*^
	T-V+	54.85	8.40	0.80	16.15	0.8765^*^
	T+V-	133.10	5.20	0.25	12.25	0.6075
	T+V+	134.55	5.45	0.25	12.35	0.6290
		**Diameter, mm**				
*C. cajan*	T-V-	4.85	2.25	0.60	2.90	0.9655^*^
	T-V+	4.85	2.25	0.60	2.90	0.9655^*^
	T+V-	1.05	1.25	−6.50	−6.80	0.5235
	T+V+	0.05	4.80e^−10^	−1.10	−0.05	0.3690
*C. ensiformis*	T-V-	5.20	3.10	1.90	9.90	0.9735^*^
	T-V+	5.05	3.65	2.70	13.65	0.9570^*^
	T+V-	1.95	5.50e^−10^	−1.15	−2.25	0.4040
	T+V+	3.35	1.10e^−12^	−5.70	−19.10	0.5220
*M. pruriens*	T-V-	4.50	2.60	1.70	7.65	0.9280^*^
	T-V+	4.40	2.65	1.65	7.25	0.9390^*^
	T+V-	3.90	2.60	1.65	6.40	0.9245^*^
	T+V+	3.30	4.25	3.15	10.40	0.9780^*^
*P. glaucum*	T-V-	53.05	17.55	1.30	68.95	0.9940^**^
	T-V+	43.20	7.25	0.80	34.55	0.9940^**^
	T+V-	62.60	4.90	0.35	21.90	0.9970^**^
	T+V+	243.05	5.55	0.15	36.45	0.9960^**^

*Significant code*:

** *p < 0.01*;

* *p < 0.05*.

In contrast, the association of tebuthiuron and vinasse allowed *C. ensiformis* to achieve its highest primary growth rate, whether specific or absolute. The negative estimation for absolute growth rate for this specie in soil with tebuthiuron alone was proof that vinasse was an effective source of nutrients to speed up the plant's growth and development, thus assisting in phytoremediation. The use of this agroindustrial residue also enhanced the height of *M. pruriens* and *P. glaucum* in soil samples with tebuthiuron. The herbicide severely limited the development of *C. cajan* and *C. ensiformis*, according to the lowest estimations for stem diameter. Thus, *M. pruriens* and *P. glaucum* are recommended over *C. cajan* and *C. ensiforms* for the phytoremediation of tebuthiuron in fields of sugarcane, even without the application of vinasse. The growth and development of *C. juncea*, the bioindicator chosen for this contaminant, became healthier and longer when sowing either *M. pruriens* and *P. glaucum*.

### Performance of *C. juncea* as Bioindicator of Phytoremediation

**Figure 3:**
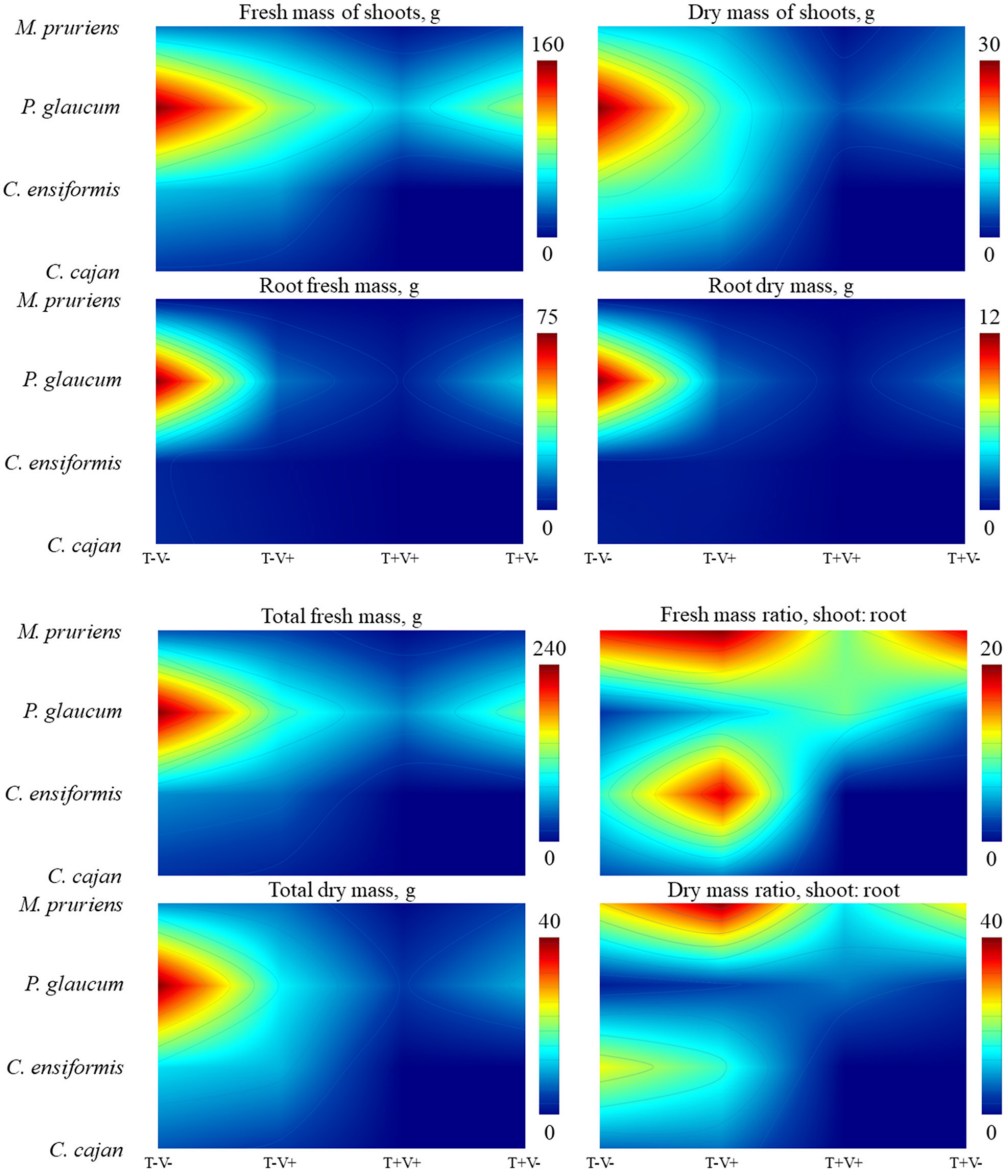
Growth and development of *C. juncea* as bioindicator of phytoremediation of green manure species in soil with tebuthiuron and vinasse.

The combination of tebuthiuron and vinasse considerably dropped the height, stem diameter, and number of leaves in *C. juncea* over time, compared to the control (Figure 3). In contrast, the herbicide alone had no significant effect, whether negative or positive, on the growth and development by the bioindicator species sowed after growing green manure species, consistent with the outcomes of explanatory analysis.

The explanation for the extensive decrease in leaves production (Table 3) may be either phytotoxicity by the compounds at high concentrations in soil or natural senescence, as plants become metabolically and physiologically ineffective over time. In contrast, the control soil sample (without tebuthiuron and vinasse) peaked in height, stem diameter, and number of leaves. Therefore, *C. juncea* was highly susceptible to tebuthiuron. Practically, this molecule more severely disabled both *C. cajan* and *C. ensiformis* to grow and develop as healthily as possible prior to sowing *C. juncea* for monitoring the potential phytoremediation of soil with green manure.

**Table 3:** Performance of *Crotalaria juncea* after green manure species cultivation in soil with tebuthiuron and vinasse.

**Species**	**Test**			
	**T-V-**	**T-V+**	**T+V-**	**T+V+**
	**Height, cm**			
*C. cajan*	74.60 Aa	60.00 Aa	ND^*^	ND^*^
*C. ensiformis*	73.00 Aa	72.20 Aa	ND^*^	ND^*^
*M. pruriens*	78.40 Aa	85.80 Aa	58.50 Aab	18.75 Ab
*P. glaucum*	72.80 Aa	66.80 Aa	57.00 Aab	11.50 Ab
	**Diameter, mm**			
*C. cajan*	4.60 Aa	4.15 Aa	ND^*^	ND^*^
*C. ensiformis*	4.45 Aa	4.25 Aa	ND^*^	ND^*^
*M. pruriens*	4.85 Aa	4.90 Aa	3.40 Aab	1.80 Ab
*P. glaucum*	4.40 Aa	3.60 Aa	2.80 Aab	1.60 Ab
	**Leaves**			
*C. cajan*	32.40 Aa	22.80 Aab	ND^*^	ND^*^
*C. ensiformis*	31.40 Aa	32.80 Aa	ND^*^	ND^*^
*M. pruriens*	32.20 Aa	34.00 Aa	24.50 Aab	10.00 Ab
*P. glaucum*	34.80 Aa	25.60 Aa	22.00 Aab	7.50 Ab
	**Shoot fresh mass, g**			
*C. cajan*	25.45 Aa	21.20 Aa	ND^*^	ND^*^
*C. ensiformis*	23.35 Aa	30.80 Aa	ND^*^	ND^*^
*M. pruriens*	29.75 Aa	34.55 Aa	12.00 Ab	2.35 Ab
*P. glaucum*	28.95 Aa	21.25 Bab	7.80 Ab	0.25 Ab
	**Shoot dry mass, g**			
*C. cajan*	22.80 Aa	13.80 Ab	ND^*^	ND^*^
*C. ensiformis*	23.95 Aa	15.65 Aa	ND^*^	ND^*^
*M. pruriens*	7.90 Aa	6.90 Aa	2.20 Aab	0.35 Ab
*P. glaucum*	6.05 Aa	3.70 Bab	1.25 Ab	0.10 Ab
	**Root fresh mass, g**			
*C. cajan*	5.20 Aa	4.30 Aa	ND^*^	ND^*^
*C. ensiformis*	7.20 Aa	5.65 Aa	ND^*^	ND^*^
*M. pruriens*	24.05 Aa	19.20 Aa	9.55 Aa	2.80 Ab
*P. glaucum*	19.10 Aab	24.50 Aa	6.70 Aab	0.10 Ab
	**Root dry mass, g**			
*C. cajan*	2.15 Aa	1.30 Aa	ND^*^	ND^*^
*C. ensiformis*	3.40 Aa	1.75 Aab	ND^*^	ND^*^
*M. pruriens*	3.00 Aa	2.55 Aa	0.90 Aab	0.15 Ab
*P. glaucum*	1.95 Aab	2.75 Aa	0.45 Abc	0.05 Ac

*Same letters, whether uppercase in the column and lowercase in the row, describe statistically similar means by post hoc Tukey's HSD test at p < 0.05;*.

* *As the plant dead, the trait became undetected (ND)*.

The behavior of bioindicator species *C. juncea* supported how persistent should be tebuthiuron in a microenvironment, regardless of vinasse application as source of nutrients to speed up the growth and development and, hence, assist green manure species in extensively remediating the herbicide. *C. cajan* and *C. ensiformis* ended up much more effectively remediating tebuthiuron and, hence, ensured the soil more suitable for the bioindicator species' growth and development (Pires et al., 2008), inconsistent with the trends in this study. Some plants are capable of highly remediating contaminants (Ferraço et al., 2017). Cultivation of *C. juncea* slightly reduced sulfentrazone concentration in soil. Bioindicators of this herbicide then grew more consistently over time, regardless of sowing density, but not as consistent as control assay (Ferraço et al., 2019).

Franco et al. (2014) reported phytoremediation benefits by *Phaseolus vulgaris* on the growth and development of *Urochloa brizantha*. Plant heigh, leaf production, and area all increased in *C. juncea* with decreasing concentration of contaminant. Thus, the longer the postcultivation is, the more probable the phytoremediation is to becoming effective.

Beans in soil with picloram at 32.00 g ha^−1^ produced low amount of dry mass due to plants' death at high pesticide concentration. However, this morphological trait increased with phytoremediation by the *Urochloa* sp. (Franco et al., 2015). Belo et al. (2016) reported similar trend for the dry mass of *P. glaucum* after phytoremediation by *C. juncea* and *C. ensiformis*. Data on phytoremediation potential of herbicides, like sulfentrazone, by *P. glaucum* are neither conclusive nor conducive to commercial application yet, and this requires further investigations (Madalão et al., 2012a,b). These references supported the major findings in this study on the negative effect of tebuthiuron on biomass accumulation in roots and shoots of green manures species. Other reliable and executable bioindicators of soil decontamination are finger millet (*Eleusine cocracana)*, for chlorimuron-ethyl and sulfumeturon-methyl (Assis et al., 2010), and cucumber for picloram (Galon et al., 2017).

### Ecotoxicity Bioassays

The principal component analysis robustly reduced the dimensionality of dataset and preserved as much interpretable variability as possible into the components, PC_I_ and PC_II_. These components, collectively, explained ~70.00% variance in ecotoxicity of soil samples on germination, growth, and development of the test-organism *L. sativa* (Table 4).

**Table 4:** Principal components into ecotoxicity bioassays in soil samples with green manure species, tebuthiuron and vinasse.

**Index/variable**	**Bartlett's test of sphericity**	
Chi-squared	104.95	
Degree of freedom	10	
*p*-value	<0.01^**^	
	**Kaiser-Meyer-Olkin test**	
	**Component**	
	**PC**_**I**_	**PC**_**II**_
Eigenvalue	2.05^*^	1.20^*^
Percentage of variance	42.75	25.45
Cumulative percentage of variance	42.75	68.20
	**Loading**	
Hypocotyl	0.45	−0.55^*^
Root	0.95^**^	−0.05
%G	0.05	0.90^**^
%R	−0.35	0.25
GI	0.95^**^	0.30
	**Contribution, %**	
Hypocotyl	9.05	24.40^*^
Root	43.45^**^	0.05
%G	0.15	63.95^**^
%R	5.50	4.35
GI	41.85^**^	7.25
	Physiological vigor	Physiological anomaly

*Significant code*:

** *p < 0.01*;

* *p < 0.05*.

**Figure 4:**
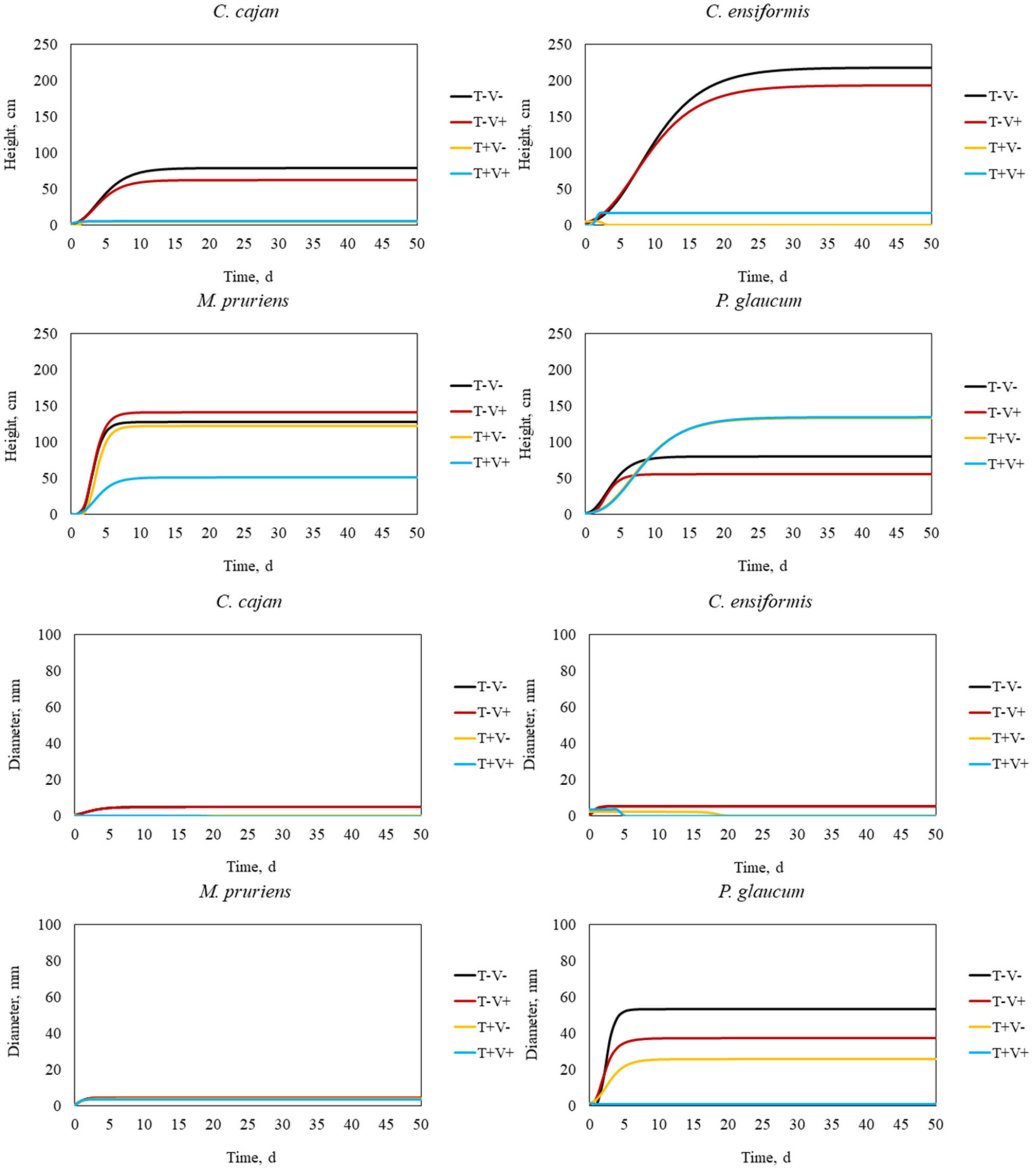
Principal components into the ecotoxicity bioassays after 50 days of cultivation of green manure species in soil with tebuthiuron and vinasse.

The first component, attributable to seed physiological vigor, had positive correlations with hypocotyl length and germination index (GI). Cartesian coordinates for soil samples with phytoremediation by either *M. pruriens* and *P. glaucum* structurally were positive in the upper right quadrant in the factorial map (Figure 4). Therefore, the more effective the tebuthiuron biodegradation is, the less probable the contaminant is to become toxic during the germination and primary growth of *L. sativa* seeds. The second component, attributable to physiological anomaly by phytotoxicity, had positive and negative loadings with germination and hypocotyl length, respectively. Soil samples with *P. glaucum* and hypocotyl were closer together in the lower right quadrant. Thus, this species should be of greater relevance to ensure plant growing and development without any severe physiological anomaly by tebuthiuron in site without vinasse.

## Conclusion

Green manure species and vinasse can remediate soils with tebuthiuron. Preliminary evidence of *M. pruriens* and *P. glaucum* show their increased capabilities of phytoremediating sites where the target herbicide exists. These species, in association with vinasse as source of soluble carbon, can decontaminate the system more effectively than *C. cajan* and *C. ensiformis*, thus enabling the bioindicator *C. juncea* to grow and develop healthier in the presence of residual tebuthiuron. As long as the manuring by fertilizing agents is effective in remediating the soil, the less probable tebuthiuron persists at high concentrations in soil and, thus, becoming harmful to non-target organisms as shown in ecotoxicity bioassays. Undergerminated seeds and severe physiological anomalies due to phytotoxicity in roots and hypocotyl of *L. sativa* are likely to decrease quickly with phytoremediation by *M. pruriens* and *P. glaucum*, which is the best chance to do this. Findings of this study are timely and should be of great importance to development and implementation of cost-effective strategies to assist in mitigating contamination of soil by tebuthiuron in sugarcane crops with vinasse application as biofertilizer. The fate of this herbicide and its potential metabolites in soil and into tissues of green manure species, especially *M. pruriens* and *P. glaucum*, is prone to scaled up designs toward an effective and safe industrial usage and could be the focus of further investigations.

## Data Availability Statement

The raw data supporting the conclusions of this article could be made available by the authors.

## Author Contributions

LF, EP, RT, EB, and PL: conceptualization. LF, RM, YF, and PL: methodology. LF, BM, RM, RV, and PL: validation. LF, BM, and PL: formal analysis, data curation, and writing—original draft. LF and YF: investigation. PL: resources, supervision, project administration, and funding acquisition. RM, EP, RV, RT, JC, EB, YF, and PL: writing—review & editing. BM, JC, and PL: visualization. All authors contributed to the article and approved the submitted version.

## Conflict of Interest

The authors declare that the research was conducted in the absence of any commercial or financial relationships that could be construed as a potential conflict of interest.
